# Engineering C_f_/ZrB_2_‐SiC‐Y_2_O_3_ for Thermal Structures of Hypersonic Vehicles with Excellent Long‐Term Ultrahigh Temperature Ablation Resistance

**DOI:** 10.1002/advs.202304254

**Published:** 2023-10-22

**Authors:** Bowen Chen, Dewei Ni, Weichao Bao, Chunjing Liao, Wei Luo, Erhong Song, Shaoming Dong

**Affiliations:** ^1^ State Key Laboratory of High Performance Ceramics and Superfine Microstructure Shanghai Institute of Ceramics Chinese Academy of Sciences Shanghai 201899 China; ^2^ Structural Ceramics and Composites Engineering Research Center Shanghai Institute of Ceramics Chinese Academy of Sciences Shanghai 201899 China; ^3^ Hangzhou Institute for Advanced Study University of Chinese Academy of Sciences Hangzhou 310024 China; ^4^ Analysis and Testing Center for Inorganic Materials Shanghai Institute of Ceramics Chinese Academy of Sciences Shanghai 201899 China; ^5^ Center of Materials Science and Optoelectronics Engineering University of Chinese Academy of Sciences Beijing 100049 China; ^6^ State Key Laboratory for Modification of Chemical Fibers and Polymer Materials College of Materials Science and Engineering Institute of Functional Materials Donghua University Shanghai 201620 China

**Keywords:** Density functional theory calculations, long‐term multi‐cycle ablation, Ceramic matrix composites

## Abstract

Ultrahigh temperature ceramic matrix composites (UHTCMCs) are critical for the development of high Mach reusable hypersonic vehicles. Although various materials are utilized as the thermal components of hypersonic vehicles, it is still challenging to meet the ultrahigh temperature ablation‐resistant and reusability. Herein, the Y_2_O_3_ reinforced C_f_/ZrB_2_‐SiC composites are designed, which demonstrates near‐zero damage under long‐term ablation at temperatures up to 2500 °C for ten cycles. Notably, the linear ablation rate of the composites (0.33 µm s^−1^) is over 24 times better than that of the conventional C_f_/C‐ZrC at 2500 °C (8.0 µm s^−1^). Moreover, the long‐term multi‐cycle ablation mechanisms of the composites are investigated with the assistance of DFT calculations. Especially, the size effect and the content of the Zr‐based crystals in the oxide layer fundamentally affect the stability of the oxide layer and the ablation properties. The ideal component and structure of the oxide layer for multi‐cycle ablation condition are put forward, which can be obtained by controlling the Y_2_O_3_/ZrB_2_ mole ratio and establishing Y‐Si‐O – *t*‐Zr_0.9_Y_0.1_O_1.95_ core‐shell nano structure. This work proposes a new strategy for improving the long‐term multi‐cycle ablation resistance of UHTCMCs.

## Graphical abstract

The Y_2_O_3_ reinforced C_f_/ZrB_2_‐SiC composites are designed in this work to form the high stability oxide protective layer. The size effect of the formed Zr_0.9_Y_0.1_O_1.95_ nano‐crystals increases the viscosity of oxide layer and retarding the evaporation of SiO_2_ phase during ablation, which significantly improve the long‐term and cyclic ablation properties of the composites.

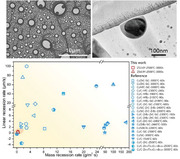

## 1 Introduction

Advanced hypersonic vehicles, which possess extremely high flight speed above Mach 5, offer significant potential benefits for military and aerospace transport applications.^[^ ^1^, ^2^, ^3^ ^]^ The development of hypersonic flight has attracted global attention, as demonstrated by the United States’ X‐51 flight with a speed of Mach 6–6.5,^[^ ^4^, ^5^ ^]^ and Russia's deployment of Kh‐47M2 hypersonic missiles with a top speed of Mach 12.^[^ ^6^ ^]^ Due to the extreme service environment, hypersonic vehicles are subjected to the scouring of dissociated air, resulting in thermal structures experiencing surface temperature exceeding 2000 °C. To address this challenge, various ablation‐resistant materials have been developed, including refractory metals,^[^ ^7^ ^]^ carbon fiber reinforced carbon matrix composites (C_f_/C),^[^ ^8^, ^9^, ^10^ ^]^ ultrahigh temperature ceramics (UHTCs),^[^ ^11^, ^12^ ^]^ etc. The next generation of hypersonic vehicles requires thermal structures to be reusable in even harsher service environments above 2400 °C, while also meeting lightweight requirements.^[^ ^13^, ^14^, ^15^ ^]^ Traditional refractory metals, C_f_/C composites and UHTCs cannot feed the demand of the next generation hypersonic vehicles.^[^ ^16^, ^17^, ^18^ ^]^ Continuous fiber reinforced ultrahigh temperature ceramic matrix composites (UHTCMCs) exhibit excellent ablation resistance due to the existence of UHTCs matrix.^[^ ^19^, ^20^, ^21^ ^]^ Fiber reinforcement improves the thermal shock resistance and reliability of UHTCMCs through bearing load and deflecting cracks.^[^ ^16^, ^22^, ^23^ ^]^ These unique properties make UHTCMCs the leading candidates for use in extreme service environment on the next generation hypersonic vehicles.^[^ ^1^, ^24^, ^25^ ^]^

The ablation‐resistant mechanisms of UHTCMCs are primarily attributed to the formation of the dense uniform oxides protective layer through the oxidation of matrix phase during ablation. Especially, the oxide layer prevents further ablation damage of UHTCMCs by resisting inward oxygen penetration.^[^ ^23^, ^26^, ^27^ ^]^ Therefore, it is critical to design the composition of matrix phase to guarantee the anti‐ablation properties of UHTCMCs. While Ta‐based UHTCMCs, such as C_f_/C‐TaC, C_f_/TaC‐SiC, have superior thermo‐mechanical properties, their melting point of Ta_2_O_5_ is limited to 1800 °C, which restricts their service temperature.^[^ ^3^, ^28^, ^29^ ^]^ Zr‐based and Hf‐based UHTCMCs are ideal for withstanding ablation at temperatures above 2400 °C.^[^ ^30^, ^31^ ^]^ Particularly, Zr‐based UHTCMCs have attracted more attention due to their low density and excellent ablation resistance. Especially, the ablation resistant temperature of the Zr‐based and Hf‐based composites can be further broadened (1600–2400 °C) by forming a dense protective ZrO_2_‐SiO_2_ and HfO_2_‐SiO_2_ layer with the addition of SiC matrix phase.^[^ ^32^, ^33^, ^34^ ^]^ The excellent properties make such composites the most potential thermal protective material for reusable hypersonic vehicles.^[^ ^35^, ^36^, ^37^, ^38^, ^39^ ^]^ However, SiO_2_ evaporates violently, and ZrO_2_/HfO_2_ grains coarsen significantly at ablation temperatures up to 2500 °C, which reduces the structural stability of the oxide protective layer.^[^ ^40^, ^41^ ^]^ Moreover, the ZrO_2_/HfO_2_ phase transition induces stress and cracks in the oxide layer during multi‐cycle ablation.^[^ ^41^ ^]^ Enhancing the microstructural stability of oxide layer is the key point to improve the reusability of Zr‐based and Hf‐based composites. Thus, it is crucial to restrict the phase transition of ZrO_2_/HfO_2_ and retard the evaporation of the SiO_2_ phase to improve the long‐term multi‐cycle ablation resistance of the composites at higher temperature. The addition of lower‐valence oxides like CaO, MgO, CeO_2_, La_2_O_3_, and Y_2_O_3_ have been verified as the effective way to stabilize the ZrO_2_/HfO_2_ grains in previous studies.^[^ ^42^ ^]^ Especially for the Y_2_O_3_, which can suppress the phase transition of ZrO_2_ more efficiently.^[^ ^43^ ^]^ Besides, Y_2_O_3_ can react with SiO_2_ glassy phase to form rare earth silicate, which reduces the evaporation of the glassy phase.^[^ ^44^, ^45^ ^]^

In this work, Y_2_O_3_ reinforced C_f_/ZrB_2_‐SiC composites were designed and fabricated using a highly efficient stepwise construction strategy to enhance the structural stability of the oxides layer. The composites were subjected to long‐term (300 s) and multi‐cycle (ten cycles) ablation tests using an air plasma flame at temperature up to 2500 °C. The resulting oxides protective layer remained structurally stable even after ten ablation cycles, leading to a linear ablation rate of 0.33 µm s^−1^, which is over 24 times better than that of the conventional C_f_/C‐ZrC at 2500 °C (8.0 µm s^−1^). The oxides layer evolution and ablation‐resistant mechanisms were also discussed with the assistance of DFT calculations. This work provides a novel approach to enhance the long‐term multi‐cycle ablation resistance of UHTCMCs.

## 2 Results and Discussion

### 2.1 Long‐Term Multi‐Cycle Ablation Performance

**Figure 1:**
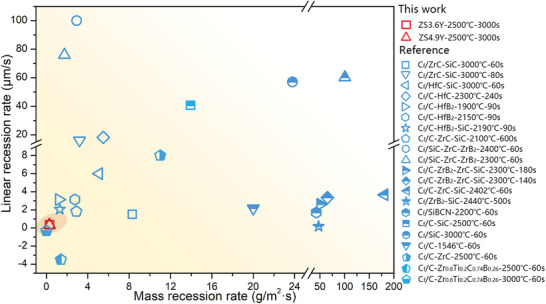
Ablation recession rates of C_f_/ZrB_2_‐SiC‐Y_2_O_3_ composites. Ablation recession rates of this work (ZS3.6Y‐2500 °C−3000s and ZS4.9Y‐2500 °C−3000s) comparing with the similar thermal structural materials including: C_f_/ZrC‐SiC‐3000 °C−60s, C_f_/SiC‐3000 °C−60s,^[^ ^47^ ^]^ C_f_/ZrC‐SiC‐3000 °C−80s,^[^ ^48^ ^]^ C_f_/HfC‐SiC‐3000 °C−60s,^[^ ^49^ ^]^ C_f_/C‐HfC‐2300 °C−240s,^[^ ^50^ ^]^ C_f_/C‐HfB_2_‐1900 °C−90s, C_f_/C‐HfB_2_‐2150°C−90s,^[^ ^51^ ^]^ C_f_/C‐HfB_2_‐SiC‐2190°C−90s,^[^ ^52^ ^]^ C_f_/C‐ZrC‐SiC‐2100°C−600s,^[^ ^21^ ^]^ C_f_/SiC‐ZrC‐ZrB_2_‐2400 °C−60s, C_f_/SiC‐ZrC‐ZrB_2_‐2300 °C−60s,^[^ ^53^ ^]^ C_f_/C‐ZrB_2_‐ZrC‐SiC‐2300 °C−180s, C_f_/C‐ZrB_2_‐ZrC‐SiC‐2300 °C−140s,^[^ ^54^ ^]^ C_f_/C‐ZrC‐SiC‐2402 °C−60s, C_f_/C‐1546 °C−60s,^[^ ^55^ ^]^ C_f_/ZrB_2_‐SiC‐2440 °C−500s,^[^ ^40^ ^]^ C_f_/SiBCN‐2200 °C−60s,^[^ ^56^ ^]^ C_f_/C‐SiC‐2500 °C−60s,^[^ ^57^ ^]^ C_f_/C‐2300 °C−30s,^[^ ^58^ ^]^ C_f_/C‐ZrC‐2500 °C−60s,^[^ ^46^ ^]^ C_f_/C‐Zr_0.8_Ti_0.2_C_0.74_B_0.26_‐2500 °C−60s, C_f_/C‐Zr_0.8_Ti_0.2_C_0.74_B_0.26_‐3000 °C−60s.^[^ ^2^ ^]^

**Figure** **1** compares the ablation recession rates of the Y_2_O_3_ reinforced C_f_/ZrB_2_‐SiC composites with other UHTCMCs reported in literature. Generally, the mass loss of the ablated samples is dominated by the release of gas oxides (CO, CO_2_, B_2_O_3_, SiO, etc.), which can also be affected by the evaporation of the SiO_2_. The linear ablation rate is calculated by determining the intrinsic thickness of the samples before and after ablation process without considering the thickness of the oxides layer, which directly reflects the damage degree of samples. Especially, higher recession rates indicate the poor ablation resistance. Herein, the composites of this work present a significant improvement of ablation resistance with near‐zero recession rates compared to the conventional UHTCMCs. The linear ablation rate of the as‐fabricated ZS4.9Y ( the sample with 4.9 vol.% Y_2_O_3_ ) composites (0.33 µm s^−1^) is over 24 times better than that of conventional C_f_/C‐ZrC (8.0 µm s^−1^) even after ten cycles of ablation at 2500 °C.^[^ ^46^ ^]^ Zeng et al. have fabricated Zr_0.8_Ti_0.2_C_0.74_B_0.26_ coated C_f_/C composites that achieve near‐zero damage during short‐term ablation at temperatures ranging from 2500 to 3000 °C. The linear ablation rate of the C_f_/C‐Zr_0.8_Ti_0.2_C_0.74_B_0.26_ is only −3.5 µm s^−1^ when considering the thickness of the oxides layer. However, the multi‐cycle ablation behavior of the C_f_/C‐Zr_0.8_Ti_0.2_C_0.74_B_0.26_ has not been studied or reported. In this work, the composites have been further modified and optimized to resist long‐term ablation at 2500 °C for ten cycles, demonstrating near zero ablation damage.

### 2.2 Design and Fabrication of C_f_/ZrB_2_‐SiC‐Y_2_O_3_ Composites

**Figure 2:**
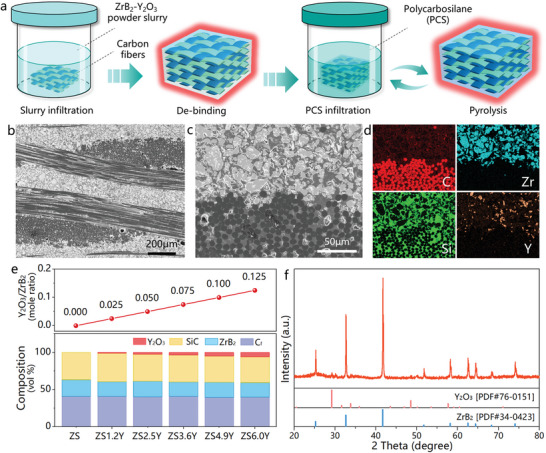
Structural and elemental composition of the C_f_/ZrB_2_‐SiC‐Y_2_O_3_ composites. a) Schematic diagram of the C_f_/ZrB_2_‐SiC‐Y_2_O_3_ composites fabrication process. b,c) Cross‐section scanning electron microscope (SEM) images of the C_f_/ZrB_2_‐SiC‐Y_2_O_3_ composites. d) EDS elemental maps of the cross‐section c.e) Y_2_O_3_/ZrB_2_ mole fraction and composites composition normalized volume fraction without porosity in different samples. f) XRD results of the sample ZS4.9Y.

A stepwise strategy was developed for preparing composites based on slurry infiltration followed by precursor infiltration and pyrolysis (PIP), as shown in **Figure** **2**a. To enhance the ablation resistance, Y_2_O_3_ reinforced C_f_/ZrB_2_‐SiC composites were fabricated, taking into account the microstructure, composition and fabrication methods of the composites. The microstructure of composites depends largely on the structure of carbon fiber preform, which needs to be considered first. During ablation, carbon fibers are oxidized, forming large holes that act as channels for inward oxygen penetration, reducing the structural stability of the oxide layer.^[^ ^35^, ^59^ ^]^ The harmfulness of the fiber oxidation becomes more pronounced with an increase in the fibers along the ablation direction (“Z” direction, Figure S1a,b, Supporting Information). To minimize the number of fibers in “Z” direction, 2D carbon fiber plain cloth (2D‐C_f_) was selected as the preform since all fibers are perpendicular to the ablation direction (Figure S1c,d, Supporting Information).

On the one hand, the fabrication process involves introducing the UHTCs matrix into carbon fiber preform. Y_2_O_3_ is used to restrict the phase transition of ZrO_2_ grains, which is the oxide of ZrB_2_ matrix. Developing a homogeneous distribution of ZrB_2_ and Y_2_O_3_ in matrix is necessary, which can be solved by slurry infiltration method.^[^ ^60^ ^]^ Firstly, ZrB_2_ and Y_2_O_3_ were ball milling mixed to form a uniform slurry. Then, the slurry was introduced into layered 2D‐C_f_ preform, followed by de‐binding to form the homogeneous ZrB_2_‐Y_2_O_3_ matrix. On the other hand, SiC matrix will be oxidized to SiO_2_ glassy phase during ablation, which can heal the cracks and pores of oxides layer.^[^ ^61^ ^]^ Carbon fibers are vulnerable under ablation environment. Therefore, the pores formed by the oxidized carbon fibers need to be healed by more SiO_2_ (Figure S1d, Supporting Information). To achieve this, it's necessary to select an appropriate fabrication method to allocate more SiC matrix around the carbon fibers. Due to the impeding of fibers, ZrB_2_‐Y_2_O_3_ powders in slurry are difficult to fill the voids in intra‐bundle area. To effectively introduce more SiC around fibers, liquid polycarbosilane (PCS) is used as the precursor of SiC ceramic, which can be introduced into the intra‐bundle area and form more SiC around fibers.^[^ ^32^, ^41^ ^]^ Therefore, PCS precursor infiltration and pyrolysis was used for the final densification of the composites. Otherwise, the ZrB_2_‐Y_2_O_3_ powders filled part of the voids, increasing the efficiency of the PIP process. Dense C_f_/ZrB_2_‐SiC‐Y_2_O_3_ composites were obtained by repeating the PIP process for five cycles.

The designed C_f_/ZrB_2_‐SiC‐Y_2_O_3_ composites possess a layered structure, where a homogeneous ZrB_2_‐SiC‐Y_2_O_3_ matrix forms in inter‐bundle area, and carbon fibers are enveloped by SiC enriching matrix. The composites show a dense matrix structure with limited pores (**Figure** 2b,c). The specific layered structure of composites makes the ZrB_2_‐SiC‐Y_2_O_3_ matrix layers serve as UHTCs coatings during ablation by forming dense oxide layer, which can effectively protect the composite from catastrophic destruction during long‐term ablation. On the other hand, no “Z” direction fibers exist in the 2D carbon fiber reinforcement, which avoids the formation of large‐scale “Z” direction oxygen diffusion channels during ablation and increases the structural stability of the oxide layer. To investigate the ablation mechanism of C_f_/ZrB_2_‐SiC‐Y_2_O_3_ composites influenced by Y_2_O_3_, composites with different Y_2_O_3_ contents were prepared. The samples with 0 to 6.0 vol.% Y_2_O_3_ have a Y_2_O_3_/ZrB_2_ mole ratio ranging from 0 to 0.125, which are labeled as ZS to ZS6.0Y, respectively (**Figure** 2e; Table S1, Supporting Information). The thermodynamic calculation indicates that the Y_2_O_3_ will react with carbon fibers at ≈2600 °C. However, the unique distribution of the Y_2_O_3_ in composites prevents the reaction. As shown in **Figure** 2b–d, the Y_2_O_3_ distribute in inter‐bundle area, and the carbon fibers are covered and protected by SiC matrix. Therefore, the fibers are isolated by SiC matrix which prevent the contact and reaction with the Y_2_O_3_ matrix. X‐ray diffraction (XRD) were utilized to further confirm the successful fabrication of C_f_/ZrB_2_‐SiC‐Y_2_O_3_ composites (**Figure** 2f), revealing ZrB_2_ as the main matrix phase with peaks at 25.2°, 32.6°, and 41.7°, while the tiny peaks at 29.2°, 48.5° corresponded to the Y_2_O_3_ matrix. Especially, the SiC matrix formed by PCS is amorphous and therefore undetectable by XRD in **Figure** 2f.^[^ ^41^ ^]^ Besides, the absence of “Z” direction fibers may slightly decrease the interlaminar shear strength which is very limited. However, due to the tight laminate structure with the thin inter layer matrix (≈200 µm), the cracks formed in matrix also can be effectively deflected by carbon fiber layer. The as‐fabricated composites still possess high mechanical performance with the interlaminar shear strength and flexural strength of 28.3 and 354.1 MPa, respectively.^[^ ^62^ ^]^

### 2.3 Long‐Term Multi‐Cycle Ablation Behavior at 2500 °C

**Figure 3:**
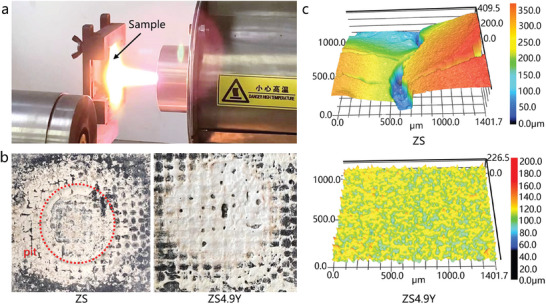
Ablation test image and morphology of the ablated surface. a) Photograph of the air plasma ablation test. b) Comparison of the ablated surface of the samples ZS and ZS4.9Y after ten ablation cycles at 2500 °C. c) 3D surface profile of the ablated surface of the samples in (b).

Long‐term multi‐cycle ablation tests were conducted to investigate the ablation performance of the C_f_/ZrB_2_‐SiC‐Y_2_O_3_ composites under realistic service conditions. To simulate high temperature environments, an air plasma flame was employed as the ablation source (**Figure** **3**a), and the ablation temperature was set as 2500 °C. Each ablation cycle lasted for 300 s, followed by air cooling for 120 s, and ten ablation cycles were performed for each sample. The main oxidation reactions of the composites during ablation include:

(1) $$C+0.5O_{2}\left(g\right)=\mathrm{CO}\left(g\right)$$

(2) $$\mathrm{SiC}+O_{2}\left(g\right)=\mathrm{SiO}\left(g\right)+\mathrm{CO}\left(g\right)$$

(3) $$\mathrm{SiC}+1.5O_{2}\left(g\right)={\mathrm{SiO}}_{2}+\mathrm{CO}\left(g\right)$$

(4) $$\mathrm{SiC}+2O_{2}\left(g\right)={\mathrm{SiO}}_{2}+{\mathrm{CO}}_{2}\left(g\right)$$

(5) $${\mathrm{ZrB}}_{2}+2.5O_{2}\left(g\right)={\mathrm{ZrO}}_{2}\left(g\right)+B_{2}O_{3}\left(g\right)$$

The changes of standard Gibbs free energy of the above reactions were calculated (Figure S2a, Supporting Information). The oxidation of ZrB_2_ become very active with the temperature reach to 2500 °C. The oxidation of C and SiC are active both in high (reaction 3, 4) and low (reaction 1, 2) oxygen partial pressure. Otherwise, the contrast ablation test methods in the manuscript are oxyacetylene or oxypropane flame tests. However, the acetylene and propane are reducing agents, which result in the ablation flame showing poor oxidation property compared with air plasma in this work.^[^ ^40^, ^63^ ^]^ Besides, the composites are designed for potential applications on hypersonic vehicles serving in near space (altitude: 20–100 km). The oxygen content drops below 6 vol.% at altitudes above 85 km. Therefore, the ablation condition used in this work is similar to the potential service environment of the composites.

**Figure** 3b,c and Figure S2b–f (Supporting Information) show the morphology of the ablation surface ablated after the 10th cycles. For the ZS sample, ablation hollows and cracks were observed on the oxide layer, and an ablation pit was found at the center of the ablated surface, indicating the exfoliation of the oxide layer. In contrast, a dense smooth oxides layer without cracks formed on the ZS4.9Y sample (**Figure** 3c). The 3D surface profile of the ablated surface revealed that the roughness of the ZS surface (82.30 µm) was ≈6.1 times higher than that of ZS4.9Y (13.45 µm). The fragmentized oxide layer resulted in a significant decrease in the multi‐cycle ablation resistance of ZS. The different structure features of the ablated surface suggest a significant increase in the structural stability and thermal shock resistance of oxides protective layer with the addition of Y_2_O_3_.

**Figure 4:**
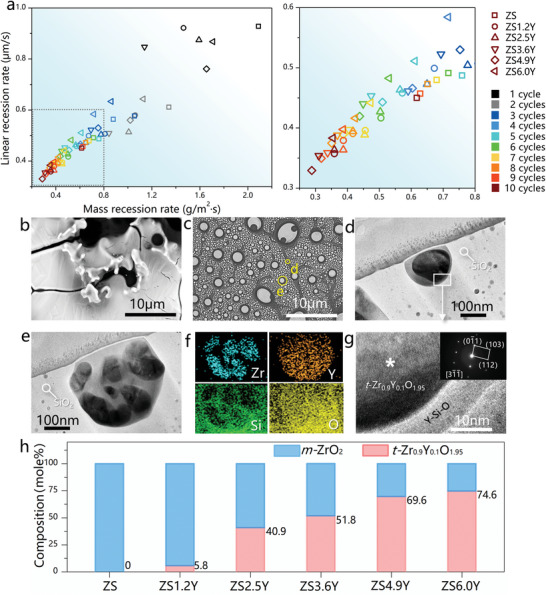
Long‐term multi‐cycle ablation behavior and microstructure of the C_f_/ZrB_2_‐SiC‐Y_2_O_3_ composites. a) Ablation recession rates of samples after each ablation cycle. b) Oxides layer surface of ZS ablated after the 10^th^ cycle. c) Oxides layer surface of ZS4.9Y ablated after the 10^th^ cycle. d,e) Cross‐section TEM micrographs of the *t*‐Zr_0.9_Y_0.1_O_1.95 –_ Y‐Si‐O core shell and agglomerated particles structure corresponding to the label in (c). f) EDS elements maps of the agglomerated particles in (e). g) High‐resolution transmission electron microscope (HR‐TEM) and selected area electron diffraction (SAED) patterns of the *t*‐Zr_0.9_Y_0.1_O_1.95 –_ Y‐Si‐O core shell structure in (d). h) *t*‐Zr_0.9_Y_0.1_O_1.95_ and *m*‐ZrO_2_ mole fraction in oxides layer of different samples.

**Figure** **4**a illustrates the ablation recession rates of C_f_/ZrB_2_‐SiC‐Y_2_O_3_ composites after each ablation cycle. Both the mass and linear ablation rates of the samples decrease and tend to stabilize with the increase of ablation cycles, indicating an increase in the protection of the formed oxides layer that remains stable over time. The C_f_/ZrB_2_‐SiC‐Y_2_O_3_ composites exhibit significantly better ablation resistance with smaller ablation rates compared to the C_f_/ZrB_2_‐SiC composite, and ZS4.9Y displays the best anti‐ablation properties after ten cycles, with a linear rate of 0.33 µm s^−1^ and a mass recession rate of 0.29 g m^−2^·s^−1^. These rates are 27% and 53% lower than those of ZS (0.45 µm s^−1^ and 0.62 g m^−2^·s^−1^), respectively. Besides, the composites experience great mass loss during 3000 s ablation process. The mass loss percentages of ZS0∼6Y samples are 14.8%, 11.7%, 9.8%, 8.0%, 6.7% and 9.5%, respectively. The mass loss is caused by the evaporation of SiO_2_, releasing of CO, CO_2_, SiO gas product during ablation which reflects the ablation resistance of the composites. The composites show less mass loss percentage after adding Y_2_O_3_, as the improvement of the anti‐ablation property. Besides, the mass loss primarily caused by the oxidation of the surface layer on the composites. The inside composites are protected by the oxide layer which experiences very limited oxidation and remains structurally intact. The composites still possess excellent mechanical properties after heat treatment at 1800 °C, with the flexural strength of ≈248.7 MPa reported in our previous work.^[^ ^41^, ^62^ ^]^

To elucidate the mechanisms by which Y_2_O_3_ affects the oxides protective layer and ablation properties, the microstructure and phase composition of the oxides layer were characterized. The oxides layer on ZS is porous with cracks, as seen in ZS1.2Y (**Figure**. 4b; Figure S3, Supporting Information). TEM and XRD results reveal that the white oxide of ZS is *m*‐ZrO_2_ twin crystal (Figures S4 and S5a, Supporting Information), and the SiO_2_ glassy phase evaporates completely, making it undetectable in the oxides layer. Cubic ZrO_2_ forms at 2500 °C and grows with the evaporation of SiO_2_ phase during ablation. During cooling, the cubic ZrO_2_ transforms into tetragonal and monoclinic phase, inducing residual stress and resulting in the formation of the twin structure in ZrO_2_ crystals and cracks among ZrO_2_ grains. Thus, the oxides layer possesses a porous structure, which tends to peel off during multi‐cycle ablation process. However, the porous structure converts to dense with the increase of Y_2_O_3_ content. Both bulk *m*‐ZrO_2_ and ZrO_2_ nano‐crystals are formed in the grey SiO_2_ glassy phase (**Figure** 4c; Figure S3c–f, Supporting Information). Due to the fast‐cooling rate of the samples, SiO_2_ remains as glassy phase that cannot be detected by XRD (Figure S5a, Supporting Information) but confirmed by EDS (**Figure** 4f; Figure S5b, Supporting Information).^[^ ^53^, ^64^ ^]^ The dense ZrO_2_‐SiO_2_ layer protects samples and reduces the ablation recession rates effectively. The results of TEM and XRD indicate that the ZrO_2_ nano‐crystal possesses a core shell structure with the *t*‐Zr_0.9_Y_0.1_O_1.95_ crystalline core and amorphous Y‐Si‐O shell (**Figure** 4d,g). The bulk *m*‐ZrO_2_ crystals are formed by the direct oxidation of large ZrB_2_ particles in matrix. In contrast, partial ZrO_2_ dissolves in SiO_2_ phase and reacts with Y_2_O_3_ to form the *t*‐Zr_0.9_Y_0.1_O_1.95_. Then, the *t*‐Zr_0.9_Y_0.1_O_1.95_ precipitates from the SiO_2_ phase as nano‐crystals during cooling and with the evaporation of SiO_2_ phase. Y_2_O_3_ stabilized ZrO_2_ alleviates the phase transition stress and prevents the formation of cracks and twin crystals.

Furthermore, the size effect and the content of the Zr‐based crystals in the oxide layer significantly affect the stability of the oxide layer and the ablation properties of the composites. The coarsen and phase transition of the *t*‐Zr_0.9_Y_0.1_O_1.95_ are prevented resulting in the forming of the *t*‐Zr_0.9_Y_0.1_O_1.95_ nano‐crystals. Such nano‐crystals distribute uniformly in the SiO_2_ glassy phase which forms the network structure preventing the evaporation of the SiO_2_ and the viscosity of the oxide layer. Consequently, the oxide layer possesses excellent structural stability and protects the under‐layer composites from further ablation damage. The Zr_0.9_Y_0.1_O_1.95_ nano‐crystals with the diameter <800 nm and the ZrO_2_ micron‐crystals with the diameter >1 µm form after adding Y_2_O_3_. The mole fractions of the crystalline in oxide layer are illustrated as **Figure** 4h, which indicates the content of Zr_0.9_Y_0.1_O_1.95_ nano‐crystals increases with the rise of Y_2_O_3_ content in composites. The Zr_0.9_Y_0.1_O_1.95_ nano‐crystals distribute uniformly in SiO_2_ glassy phase. The size effect of the nano‐crystals increases the viscosity of oxides layer and retarding the evaporation of SiO_2_ phase (Figure S6, Supporting Information), which results in increased structural stability of the oxide layer. Moreover, the mass recession rates also decrease due to the lower evaporation of SiO_2_, and as a result, the C_f_/ZrB_2_‐SiC‐Y_2_O_3_ composites are effectively protected by the dense oxide layer and exhibit improved ablation resistance. Especially, the protection of the oxides layer is also associated with the oxygen permeability of oxides. Y^3+^ induces in the formation of oxygen vacancies in Zr_0.9_Y_0.1_O_1.95_, which is beneficial for the oxygen permeability of Zr_0.9_Y_0.1_O_1.95_ compared with ZrO_2_.^[^ ^65^, ^66^ ^]^ The Zr_0.9_Y_0.1_O_1.95_ crystals serve as an oxygen diffusion medium. Excessive Zr_0.9_Y_0.1_O_1.95_ crystals enhance the oxygen permeability of oxide layer, leading to the increased linear and mass recession rates of ZS6.0Y. Comprehensively considering the advantages of Y_2_O_3_ and disadvantages of Zr_0.9_Y_0.1_O_1.95_, the C_f_/ZrB_2_‐SiC‐Y_2_O_3_ composites exhibit optimal anti‐ablation properties when the mole fraction of Zr_0.9_Y_0.1_O_1.95_ nano‐crystals is set at approximately 50–70%, which can be obtained by controlling the Y_2_O_3_/ZrB_2_ mole ratio at 0.075–0.1 during preparation.

**Figure 5:**
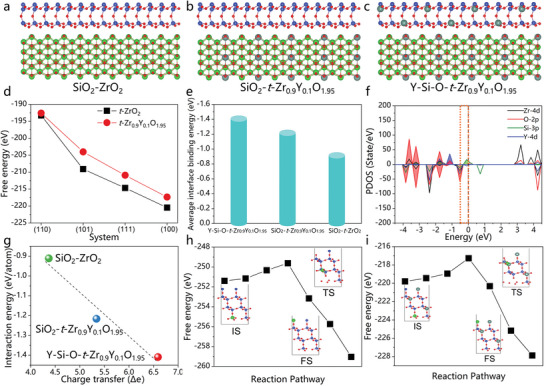
Calculated interaction energy and electronic states of multiple interfaces between SiO_2_ and *t*‐ZrO_2_, SiO_2_ and *t*‐Zr_0.9_Y_0.1_O_1.95_, Y‐Si‐O, and *t*‐Zr_0.9_Y_0.1_O_1.95_ system. a–c) The optimized structure of interfaces: SiO_2_‐*t*‐ZrO_2_, SiO_2_‐*t*‐Zr_0.9_Y_0.1_O_1.95_, Y‐Si‐O‐*t*‐Zr_0.9_Y_0.1_O_1.95_ system. (The blue, red, olive green and green balls indicate the Si, O, Y and Zr atoms around the interface, respectively.) d) The correspondingly calculated free energy of different surfaces in *t*‐ZrO_2_ and *t*‐Zr_0.9_Y_0.1_O_1.95_ system. e) The average interaction binding energy Δ*E* in different interface between SiO_2_ and ZrO_2_ system. f) Projected density of state (PDOS) of Zr‐4d, O‐2p, Si‐3p, and Y‐4d orbital of Y‐Si‐O‐*t*‐Zr_0.9_Y_0.1_O_1.95_ system. g) Linear correlation between Δ*E* and the amount of charge transfer of SiO_2_ in different ZrO_2_ surface. The calculated diffusion energy barrier of Zr^4+^ in the h) SiO_2_ and i) Y‐Si‐O system. The insets are the structure of the corresponding IS (initial state), TS (transition state) and FS (final state). The Y‐Si‐O system reveals the higher diffusion energy barrier for Zr atom diffusion, further suppressing the precipitation of Zr atom.

To reveal the formation mechanisms of *t*‐Zr_0.9_Y_0.1_O_1.95_ nano‐crystals and the core‐shell structure, density functional theory DFT calculations were carried out to further investigate the interfacial binding properties. Multiple interfaces, including SiO_2_‐*t*‐ZrO_2_, SiO_2_‐*t*‐Zr_0.9_Y_0.1_O_1.95_ and Y‐Si‐O – *t*‐Zr_0.9_Y_0.1_O_1.95_, were constructed and their relaxed structures were compared (**Figure** **5**a–c). In addition, the inherent wrinkle and distorted outmost wall of SiO_2_ is due to the interaction between SiO_2_ and ZrO_2_ (100) surfaces. The corresponding calculated free energy of four different surface structures were also represented (**Figure** 5d), qualitatively reflecting the most stable structures such as ZrO_2_‐(100) and *t*‐Zr_0.9_Y_0.1_O_1.95_‐(100) at the interfaces. It can be seen that the free energy of ZrO_2_‐(100)/*t*‐Zr_0.9_Y_0.1_O_1.95_‐(100) is larger than that of other surfaces, owing to the difference in electronegativity of the interface. As shown in **Figure** 5e, the average interaction binding energy (Δ*E*) values for three types of interfaces ranged from −0.911 to −1.409 eV/atom. Particularly, the Y‐Si‐O surface exhibits a stronger bonding strength of ≈−1.409 eV/atom when connected with the active Y terminated ZrO_2_‐(100) surface, and the Δ*E* of Y‐Si‐O – *t*‐Zr_0.9_Y_0.1_O_1.95_ is apparently larger than that of SiO_2 –_ *t*‐Zr_0.9_Y_0.1_O_1.95_ and SiO_2 –_ *t*‐ZrO_2_ system. Thus, it is deduced that the stronger interaction of interface is beneficial to form a Y‐Si‐O – *t*‐Zr_0.9_Y_0.1_O_1.95_ core‐shell structure and inhibit the coarsening of *t*‐Zr_0.9_Y_0.1_O_1.95_ grains. As shown in **Figure** 5f, the project of density of states for Y‐Si‐O – *t*‐Zr_0.9_Y_0.1_O_1.95_ interface is revealed to further understand the enhancement mechanism of interface. In fact, the value of electronic states near Fermi level (−0.5 to 0 eV highlighted by orange rectangular areas) determines the interaction energy of interface. It is found that density of state for O‐2*p* in free SiO_2_ is mainly below the Fermi level. Thereby, the electron states of O‐2*p* orbital in free Y‐Si‐O around Fermi level preferentially hybridize with Y‐4*d* and Zr‐4*d* orbitals in free *t*‐Zr_0.9_Y_0.1_O_1.95_ in order to form the interface, inducing more extra free electrons transfer and thus enhanced strength of Y‐O bond. The other electron states of O‐2p orbitals in free Y‐Si‐O between −0.5 and 0 eV are dragged to the deep energy level (−4–3 eV) due to hybridizing with the Si‐3*p*, Zr‐4*d* orbitals in free *t*‐Zr_0.9_Y_0.1_O_1.95_. After interface formation, the main electron states of O‐2*p*, Y‐4*d*, Zr‐4*d*, orbital at Y‐Si‐O‐*t*‐Zr_0.9_Y_0.1_O_1.95_ interface apparently disappear around Fermi level, which facilitates the charge distribution. Meanwhile, the formation of Y‐O bond can also promote thermodynamic stability of interface. For the Zr atom precipitation process in SiO_2_ and Y‐Si‐O system, it may introduce an additional energy barrier and govern the overall reaction pathway. From the kinetic viewpoints, the diffusion barriers of Zr atom play a key role in suppressing/enhancing the Zr precipitation process. The considerably slow rate of the Zr diffusion has greatly hindered the overall precipitation process. The diffusion energy barrier of Zr atom in the SiO_2_ and Y‐Si‐O system was calculated as shown in **Figure** 5h,i. The results indicate that the diffusion of Zr in the Y‐Si‐O system exhibits relative higher diffusion barriers (2.53 eV), comparing to that in the SiO_2_ system (1.77 eV). Therefore, the *t*‐Zr_0.9_Y_0.1_O_1.95_ grains are isolated by Y‐Si‐O phase in agglomerated particles (**Figure** 4e,f), and the growth of *t*‐Zr_0.9_Y_0.1_O_1.95_ crystals is restricted due to the high diffusion energy of Zr in the Y‐Si‐O system and stronger interaction of Y‐Si‐O – *t*‐Zr_0.9_Y_0.1_O_1.95_. Overall, the results of DFT calculations provide the guidance of the structural design for oxides layer, which is of significance for the further studies on reusable UHTCMCs.

## 3 Conclusion

In this work, we have developed Y_2_O_3_ reinforced 2D‐C_f_/ZrB_2_‐SiC composites through a structural and composition design approach, aimed at improving the long‐term multi‐cycle ablation resistance of UHTCMCs. An efficient stepwise fabrication strategy was employed to obtain composites with a uniform distribution of the matrix phase. Our results demonstrate excellent long‐term anti‐ablation performance at 2500 °C for ten cycles, with nearly zero ablation damage observed. The linear ablation rate of our composites (0.33 µm ^−1^s) is over 24 times better than that of conventional C_f_/C‐ZrC at 2500 °C (8.0 µm ^−1^s). It also proved insights into the oxides layer evolution and ablation mechanisms of the composites, assisted by DFT calculations. The Y‐Si‐O – *t*‐Zr_0.9_Y_0.1_O_1.95_ core‐shell structure forms in oxides protective layer due to their stronger interaction binding energy. The higher Zr^4+^ diffusion barrier of Y‐Si‐O significantly inhibits the precipitation of the *t*‐Zr_0.9_Y_0.1_O_1.95_ nano‐crystals. Furthermore, the size effect and the content of the Zr‐based crystals in the oxide layer fundamentally affect the stability of the oxide layer and the ablation properties of the composites. And the ideal component of the oxide layer for multi‐cycle ablation condition is put forward. Besides, this work has demonstrated that an effective approach to maintaining the structural stability of oxide protective layer is to reduce the positive ions migration rate, impede the phase transition of the high melting point oxides, increase the viscosity of glassy phase and maintain the structural stability of the oxides layer. These methods are key to improving the ultrahigh temperature multi‐cycle ablation resistance of UHTCMCs.

## 4 Experimental Section

### Material and Preparation

An efficient stepwise fabrication strategy is proposed for preparing the Y_2_O_3_ reinforced C_f_/ZrB_2_‐SiC composites with a uniform distribution of the matrix phase. The T300 2D plain carbon fiber cloth was used as reinforcement for improving the fabrication efficiency and alleviate the oxidation of the composites. The fiber cloth was first coated with pyrocarbon interface by chemical vapor infiltration (CVI). Then, eight layers of fiber cloth were stacked as the preform. ZrB_2_‐Y_2_O_3_ slurry was mixed by ball milling at 150 r min^−1^ for 6 h. The volume fraction of ZrB_2_ (1–3 µm) and Y_2_O_3_ (1–3 µm) powders were set on the basis of Table S1 (Supporting Information). Phenolic resin and ethyl alcohol were used as the dispersant and solvent. The weight fraction of ZrB_2_‐Y_2_O_3_ powders in slurry is 60%. And the weight of phenolic resin is 5% of that of ZrB_2_‐Y_2_O_3_ powders. The slurry was introduced into fiber preform by vacuum infiltration method. The C_f_/slurry was dried at 100 °C for 6 h and then debinding at 600 °C for 1 h to form a porous C_f_/ZrB_2_‐Y_2_O_3_. Finally, C_f_/ZrB_2_‐SiC‐Y_2_O_3_ composites were obtained by PCS infiltration and pyrolysis at 1100 °C for 1 h. During pyrolysis process, the PCS precursor converts to amorphous SiC and fills the void in the C_f_/ZrB_2_‐Y_2_O_3_. To reach the dense composites, the PIP process was repeated until the weight increase <1%.

### Ablation Tests

The ablation behavior of the C_f_/ZrB_2_‐SiC‐Y_2_O_3_ samples were evaluated by air plasma flame (QHKJ‐DLZ50, Beijing Qinhe Science and Technology Co. Ltd, Beijing, China) with the nozzle diameter of 10 mm (Figure 3a). Air and nitrogen were used as working gas with the flux of 0.5 and 2.7 m^3^ h^−1^, respectively. The surface temperature of the samples was determined by a bichromatic infrared pyrometer which was stabilized at 2500 °C by automatically controlling the distance between sample surface and plasma nozzle. The ablation process was carried out at 2500 °C for 300 s followed by air cooling for 120 s during each ablation cycle. The samples were processed into 30 mm × 45 mm × 4 mm for the ablation tests. The linear (LAR) and mass (MAR) ablation recession rates were calculated according to *LAR* = Δ*l*/Δ*t* and *MAR* = Δ*m*/(*S*·Δ*t*), respectively, where Δ*l* and Δ*m* are the thickness decrease and mass loss of samples after ablation; *S* is the surface area of the ablation zone; Δ*t* is the whole ablation time.

### Characterization Methods

A field emission scanning electron microscope (Thermao Fisher Verios G4 UC) was employed to observe the morphology of samples. High resolution transmission electron microscope (HR‐TEM) images and selected area electron diffraction (SAED) patterns and energy‐dispersive X‐ray spectroscopy (EDS) were obtained by spherical aberration field emission transmission electron microscope (Hitachi HF5000). TEM samples were prepared by focused ion beam (FIB, FEI Versa 3D). Phase compositions of the composites before and after the ablation test were determined by X‐ray diffraction (XRD, Ultima, IV, Rigaku Corporation, Kyoto, Japan) using Cu‐Kα radiation. For phase analysis, the 2θ range of the scan was set as 10–80°, with the resolution of 0.05°. For the phase content determination, the scanning resolution was 0.02° with the speed of 0.5°/min. The oxide layer was crushed and grinded to micron powder for XRD analysis. The results were analyzed by MDI Jade software to calculate the *m*‐ZrO_2_ and *t*‐Zr_0.9_Y_0.1_O_1.95_ phase content based on the “Value K” method. The thickness of the samples was measured by ultra‐depth optical microscope at the cross‐section of ablation area center. The open porosity of the composites was measured by the Archimedes’ method using deionized water as immersing medium. The 3D surface profile and roughness of the ablated surface were conducted by the confocal laser scanning microscope (KEYENCE, VK‐X110).

### Computational Methods

All density functional theory (DFT) calculations are performed in the Vienna Ab‐initio simulation package (VASP) .^[^ ^67^, ^68^ ^]^ The generalized gradient approximation (GGA) with the Perdew‐Burke–Ernzerhof exchange‐correlation functional and a 400 eV cutoff for the plane‐wave basis set are employed.^[^ ^69^ ^]^ The projector‐augmented plane wave (PAW) was used to describe the electron‐ion interactions.^[^ ^70^ ^]^ All the calculations were spin‐polarized and the convergence threshold was set as 10^−4^ eV in energy and 0.05 eV Å^−1^. The k‐point sampling of the Brillouin zone was obtained using a 2×2×1 by the Monkhorst–Pack scheme. Denser k‐points (4×4×1) were used for the electronic structure calculations. The vacuum slab of 15 Å was inserted in the z‐direction for surface isolation to eliminate periodic interaction. For the systems the interaction energy ∆*E* of the interface between the Y‐Si‐O and *t*‐Zr_0.9_Y_0.1_O_1.95_ is defined as:

(6) $$\Delta E\;=\;\frac{\left[E_{\mathrm{total}}-E_{Y-\mathrm{Si}-O}-E_{t-Zr_{0.9}Y_{0.1}O_{1.95}}\;\right]}{N}$$

where the subscripts total, Y‐Si‐O and *t*‐Zr_0.9_Y_0.1_O_1.95_ and N denote the total energies, the energies of Y‐Si‐O, *t*‐Zr_0.9_Y_0.1_O_1.95_ and the number of atoms of the interfaces.

## Conflict of Interest

The authors declare no conflict of interest.

## Supporting information

Supporting Information

## Data Availability

The data that support the findings of this study are available from the corresponding author upon reasonable request.
