# Supplementary material for: Engineering Cf/ZrB2‐SiC‐Y2O3 for Thermal Structures of Hypersonic Vehicles with Excellent Long‐Term Ultrahigh Temperature Ablation Resistance
Source: Adv Sci (Weinh). 2023 Oct 22;10(34):2304254. doi: 10.1002/advs.202304254 (PMC10700681; doi:10.1002/advs.202304254)
Supplement: Supplementary file 1 — Supporting Information [file ADVS-10-2304254-s001.pdf]

ADVANCED SCIENCE
Open Access

Supporting Information

for *Adv. Sci.*, DOI 10.1002/advs.202304254

Engineering C_f/ZrB_2-SiC-Y_2O_3 for Thermal Structures of Hypersonic Vehicles with Excellent Long-Term Ultrahigh Temperature Ablation Resistance

Bowen Chen, Dewei Ni, Weichao Bao, Chunjing Liao, Wei Luo*, Erhong Song* and Shaoming Dong**

Supporting Information

Engineering C_f/ZrB_2-SiC-Y_2O_3 for thermal structures of hypersonic vehicles with excellent ultra-high temperature ablation resistance and reusability

Bowen Chen[a, b], Dewei Ni[a, b, *], Weichao Bao[a, c], Chunjing Liao[a, b], Wei Luo[e, *], Erhong Song[a, *], Shaoming Dong[a, b, d, *]

[a] State Key Laboratory of High Performance Ceramics and Superfine Microstructure, Shanghai Institute of Ceramics, Chinese Academy of Sciences, Shanghai, China

[b] Structural Ceramics and Composites Engineering Research Center, Shanghai Institute of Ceramics, Chinese Academy of Sciences, Shanghai, China

[c] Analysis and Testing Center for Inorganic Materials, Shanghai Institute of Ceramics, Chinese Academy of Sciences, Shanghai, China

[d] Center of Materials Science and Optoelectronics Engineering, University of Chinese Academy of Sciences, Beijing, China

[e] State Key Laboratory for Modification of Chemical Fibers and Polymer Materials, College of Materials Science and Engineering, Donghua University, Shanghai, China

Corresponding authors: deweini@mail.sic.ac.cn (Dewei Ni); ehsong@mail.sic.ac.cn (Erhong Song); wluo@dhu.edu.cn (Wei Luo); smdong@mail.sic.ac.cn (Shaoming Dong)

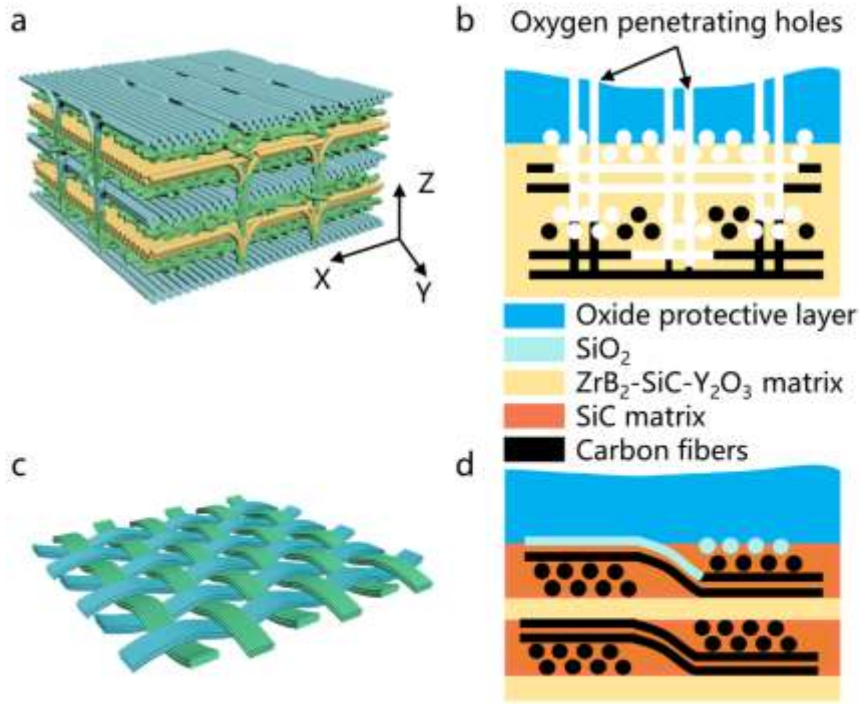

Figure S1. Schematic of ablated UHTCMCs cross-section structure influenced by different fiber preform. **a** Three-dimensional carbon fiber preform with "Z" direction fibers. **b** "Z" direction fibers in UHTCMCs are oxidized to form the oxygen penetrating holes and porous oxide layer during ablation. **c** Two-dimensional carbon fiber plain cloth with fibers along "X" and "Y" directions. **d** The C_f/ZrB_2-SiC-Y_2O_3 composites with layered structure tend to form a dense oxide layer during ablation. More SiC distributes in fiber layer which forms adequate SiO_2 glassy phase to fill up the holes generated by oxide fibers.

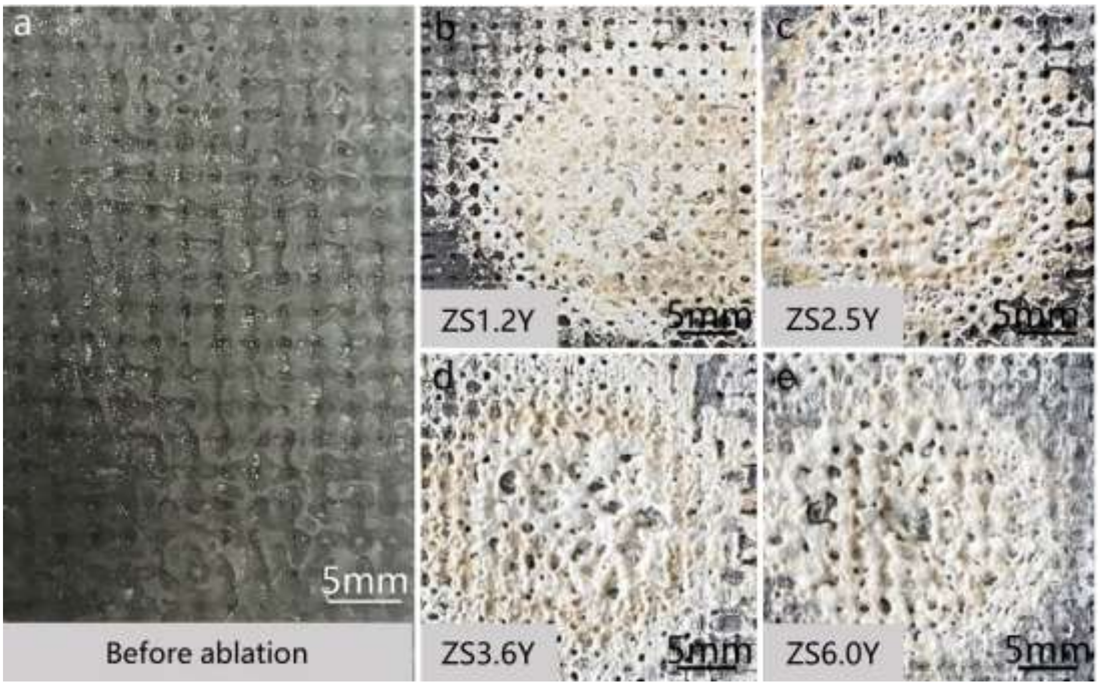

Figure S2. Morphology of samples. a before ablation test. Ablated surface of the samples after the 10th ablation: **b** ZS1.2Y, **c** ZS2.5Y, **d** ZS3.6Y, **e** ZS6.0Y.

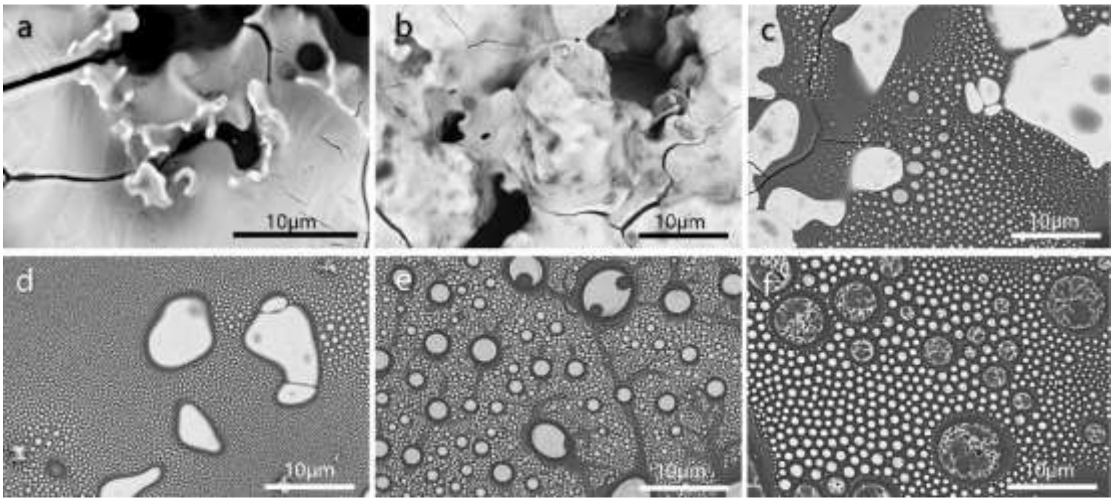

Figure S3. Oxide layer surface microstructure after the 10th ablation cycle: a ZS, **b** ZS1.2Y, **c** ZS2.5Y, **d** ZS3.6Y, **e** ZS4.9Y, **f** ZS6.0Y.

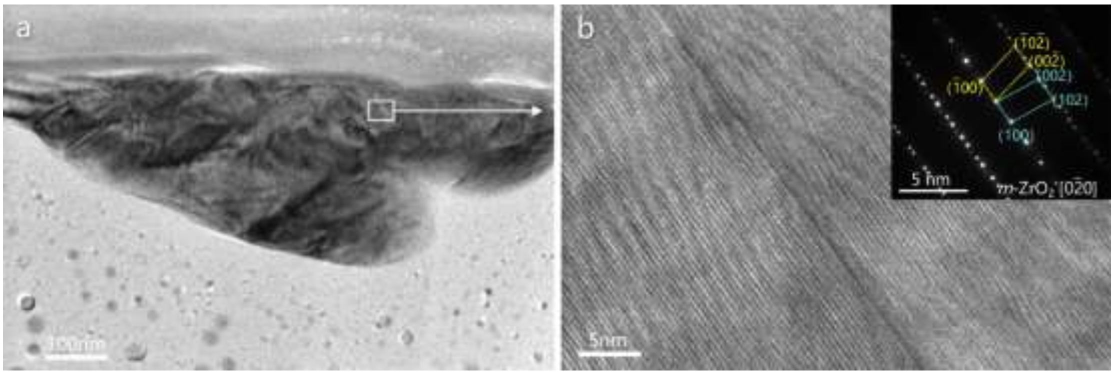

Figure S4. Microstructure and crystal structure of ZrO_2 in ZS. a TEM micrograph of the *m*-ZrO_2 in ZS oxide layer. **b** HR-TEM image and SAED patterns of the *m*-ZrO_2. *m*-ZrO_2 possesses the twin structure.

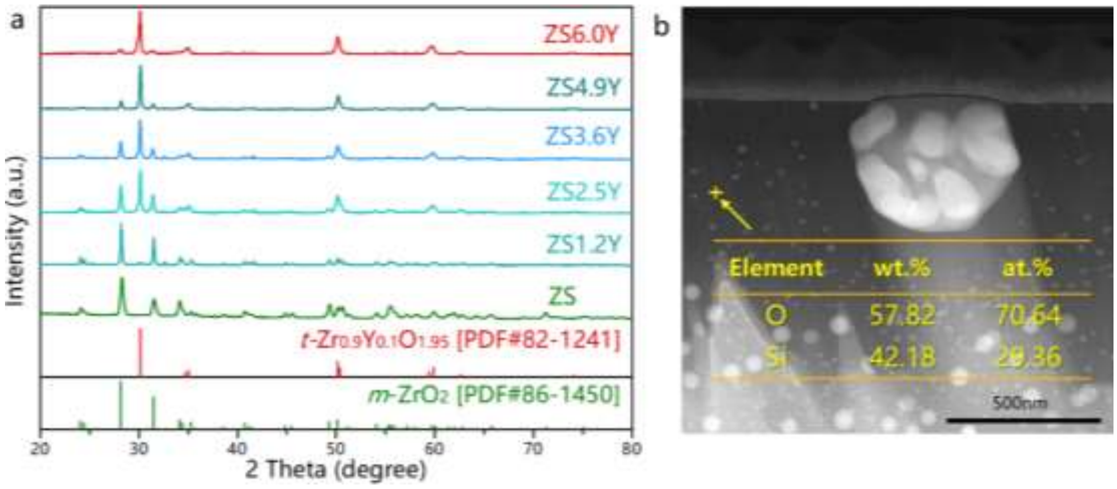

Figure S5. Phase composition of the oxide layer. a XRD results of the oxide layer after the 10th ablation cycle. With the increase of Y_2O_3 content, the peaks of *t*-$Zr_{0.9}Y_{0.1}O_{1.95}$ become more clear compare with that of *m*-ZrO_2. **b** High-angle annular dark-field scanning transmission electron microscopy (HAADF-STEM) image and corresponding EDS results of the cross-section of ZS4.9Y oxide layer.

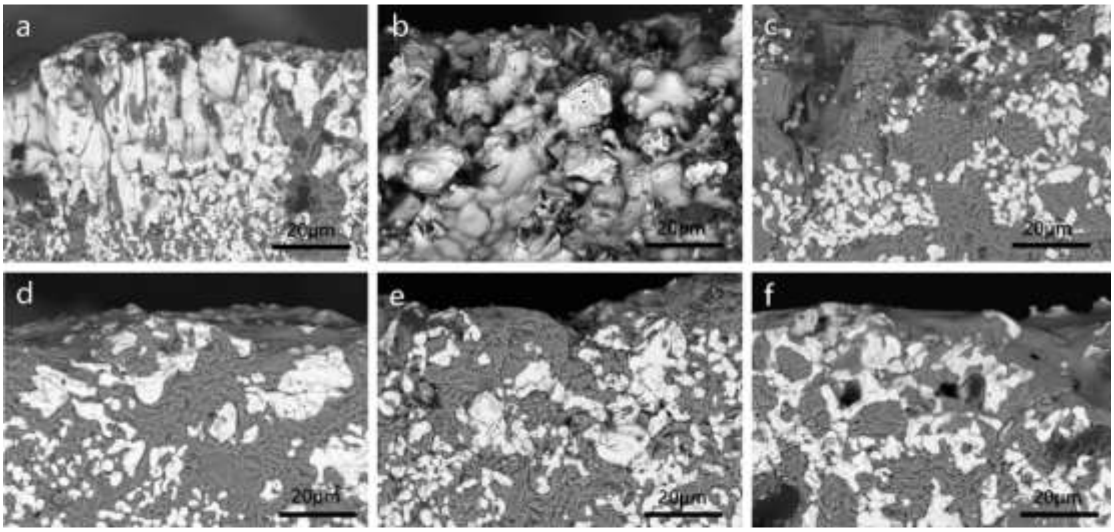

Figure S6. Cross-section microstructure of oxide layer after the 10th ablation cycle: a ZS, **b** ZS1.2Y, **c** ZS2.5Y, **d** ZS3.6Y, **e** ZS4.9Y, **f** ZS6.0Y.

Table S1. Volume fraction of the ZrB_2, Y_2O_3, SiC and C_f in different samples.

Component	Vol%					
	ZS	ZS1.2Y	ZS2.5Y	ZS3.6Y	ZS4.9Y	ZS6.0Y
ZrB_2	22.2	19.7	20.9	19.5	20.3	19.6
Y_2O_3	-	1.2	2.5	3.6	4.9	6.0
SiC	36.8	38.3	36.3	36.0	35.2	34.6
C_f	41.0	40.8	40.3	40.9	39.6	39.8

Table S2. Ablation rate of the composites in Figure 1.

Material	Ablation temperature (°C)	Total ablation time (s)	MAR (g/m^2·s)	LAR (μm/s)	Ablation cycles	Ref.
ZS3.6Y	2500	3000	0.31	0.35	10	This work
ZS4.9Y	2500	3000	0.29	0.33	10	This work

C_f/ZrC-SiC	3000	60	8.3	1.5	1	Ceram. Int. 42 (2016) 19019-19026
C_f/ZrC-SiC	3000	80	3.2	16	1	Corros. Sci. 86 (2014) 131-141
C_f/HfC-SiC	3000	60	5.1	6	1	J. Alloy. Compd. 739 (2018) 955-960
C_f/SiC	3000	60	23.8	57	1	Ceram. Int. 42 (2016) 19019-19026
C_f/C-$Zr_{0.8}Ti_{0.2}C_{0.74}B_{0.26}$	3000	60	0	-0.32	1	Nat. Commun. 8 (2017)
C_f/C-$Zr_{0.8}Ti_{0.2}C_{0.74}B_{0.26}$	2500	60	1.4	-3.5	1	15836
C_f/C-SiC	2500	60	13.94	40.611	1	J. Eur. Ceram. Soc. 40 (2020) 651-659
C_f/C-ZrC	2500	60	11.0	8.0		Carbon 54 (2013) 300-309
C_f/ZrB_2-ZrC	2440	500	47.2	0.18	1	Corros. Sci. 190 (2021) 109706
C_f/C-ZrC-SiC	2402	60	180.92	3.67	1	Corros. Sci. 102 (2016) 84-92
C_f/SiC-ZrC-ZrB_2	2400	60	2.92	100	1	Corros. Sci. 134 (2018)
C_f/SiC-ZrC-ZrB_2	2300	60	1.73	76	1	49-56
C_f/C-HfC	2300	240	5.5	18.21	1	Corros. Sci. 94 (2015) 165-170
C_f/C-ZrB_2-ZrC-SiC	2300	180	50.9	2.61	1	Corros. Sci. 98 (2015)
C_f/C-ZrB_2-ZrC-SiC	2300	140	65.2	3.35	1	551-559
C_f/SiBCN	2200	60	42.70	1.70	1	J. Eur. Ceram. Soc. 41 (2020) 1114-1120
C_f/C-HfB_2-SiC	2190	90	1.29	2.06	1	Mater. Design. 132 (2017) 552-558
C_f/C-HfB_2	2150	90	2.75	3.14	1	J. Alloy. Compd. 742 (2018) 123-129
C_f/C-ZrC-SiC	2100	600	2.9	1.82	1	Corros. Sci. 162 (2020) 108200
C_f/C-HfB_2	1900	90	1.21	3.14	1	J. Alloy. Compd. 742 (2018) 123-129
C_f/C	1546	60	20.009	2.16	1	Corros. Sci. 102 (2016) 84-92
